# A comparison of the da Vinci Xi vs. da Vinci Si surgical systems for radical prostatectomy

**DOI:** 10.1186/s12893-021-01406-w

**Published:** 2021-11-30

**Authors:** Kun-Yang Lei, Wen-Jie Xie, Sheng-Qiang Fu, Ming Ma, Ting Sun

**Affiliations:** Department of Urology, The First Affiliated Hospital of Nanchang University, Nanchang, 330000 Jiangxi China

**Keywords:** Da Vinci Si, Da Vinci Xi, Radical prostatectomy

## Abstract

**Background:**

To compare the perioperative and short-term efficacy and cost of the da Vinci Xi and da Vinci Si surgical systems for radical prostatectomy.

**Methods:**

We retrospectively analyzed the clinical data of 175 patients with prostate cancer who underwent radical prostatectomy with the da Vinci Si or Xi surgical systems in our hospital from June 2019 to June 2020. Of the 175 patients, 82 underwent robot-assisted laparoscopic radical prostatectomy with the da Vinci Xi surgery system, and 93 patients underwent robot-assisted laparoscopic radical prostatectomy with the da Vinci Si surgical system. The perioperative outcomes, short-term efficacy and costs were compared between the two groups.

**Results:**

The anesthesia time, operation time, docking time, indwelling catheter time and postoperative bed rest time in the Xi group were shorter than those in the Si group (respectively, 268.8 min vs. 219.3 min, P = 0.001; 228.2 min vs. 259.6 min, P < 0.001; 7.4 min vs. 12.7 min, P < 0.001; 8.6 d vs. 9.7 d, P = 0.036; 2.2 d vs. 2.6 d, P = 0.002). However, the total cost of hospitalization and the cost of intraoperative consumables in the Xi group were higher than those in the Si group (84,740.7 vs. 76,739.1 ¥, P = 0.003; 13,199.4 vs. 10,823.0 ¥, P = 0.019).

**Conclusions:**

Although the cost of robot-assisted radical prostatectomy is higher, compared with the Si system, the Xi system has better perioperative outcomes and can provide similar short-term efficacy and oncology outcomes.

## Introduction

As the most common malignant tumor in men, prostate cancer is a serious health threat [1]. There are many treatments for prostate cancer, including castration therapy, endocrine therapy, radiotherapy, chemotherapy and surgery. At present, radical prostatectomy is considered the best method for the treatment of localized prostate cancer, especially for patients with a life expectancy of more than 10 years [2]. Compared to open radical prostatectomy, traditional laparoscopic surgery has less blood loss and fewer complications but also has many shortcomings, such as the limited range of surgical instruments. To compensate for the deficiency of traditional laparoscopic surgery, a robot-assisted surgery system was developed.

At present, the worldwide mainstream da Vinci Si robotic surgical system has been favored by the majority of urologists because of its good vision, flexible robotic arms and excellent oncology outcomes [3]. Although the Si system has many advantages over laparoscopy, with the continuous improvement of urologists' requirements for surgical instruments, its shortcomings are gradually being magnified, such as the long docking time, easy friction between robotic arms, and lack of tactile feedback. Therefore, the Si system is currently in the process of being phased out and replaced by the latest Xi system produced in 2014 [4]. Currently, a small number of surgeons around the world have begun to use the Xi system for surgery and to compare it with the Si system [5, 6]. Whether the Xi system has obvious advantages compared to the Si system remains to be answered. At present, few studies have compared the Xi and Si systems, and most are retrospective investigations with a small number of samples. Therefore, there is an urgent need for more relevant evidence to help draw objective conclusions on this issue.

In this paper, we will discuss in detail the perioperative outcomes, short-term efficacy and cost of robot-assisted laparoscopic radical prostatectomy with the da Vinci Xi system in the treatment of prostate cancer compared with the Si system.

## Methods

We retrospectively analyzed the data of all patients who underwent robot-assisted radical prostatectomy with the Xi surgical system or Si surgical system in our hospital from June 2019 to June 2020. A total of 175 patients were included, including 82 patients in the Xi group and 93 patients in the Si group. All operations were performed by four urologists in our center who have been using the Si surgical system to perform urological operations since 2014; therefore, all surgeons had a wealth of experience in using robot systems to perform such operations. The acquisition of patient data was carried out with the approval of the Ethics Committee of the First Affiliated Hospital of Nanchang University. Findings are reported in accordance with STROBE guidelines.

The basic surgical procedures of the Xi group and the Si group were roughly the same, and all surgeries were carried out using the transperitoneal technique. Patients diagnosed by biopsy underwent surgery 6–8 weeks after biopsy to reduce the difficulty of the operation and minimize postoperative complications. Lymph node dissection was performed for patients considered moderate and high risk. Nerve-preserving surgery was performed without affecting the best possible surgical outcomes. The main difference between the two systems lies in the different docking processes: because there is no positioning device in the Si system, the positioning mainly depends on the subjective feelings of surgeons and nurses in the operating room. The method adopted in our hospital is that the surgical assistant uses suction as the guide, and the nurse in the operating room introduces the trolley under the guidance of the surgical assistant and then completes the docking. The Xi system uses a laser positioning system, which is more accurate than the above docking method; its robot arm is suspended from the overhead boom, and the boom can be rotated directly to the desired position when in use.

Patient data were collected, including age, body mass index (BMI), history of hypertension and diabetes, history of abdominal or pelvic surgery, American Society of Anesthesia (ASA) score, preoperative prostate-specific antigen (PSA) value, prostate volume, biopsy Gleason score, clinical T stage, D’Amico risk classification, anesthesia time, operation time, docking time, bladder neck-urethral anastomosis time, estimated blood loss, intraoperative blood transfusion volume, postoperative bed rest time, postoperative recovery time of intestinal function, postoperative pelvic drainage duration, indwelling catheter time, postoperative hospital stay duration, postoperative complications, positive surgical margin (PSM), urethral stricture, postoperative continence in the first, third and sixth months, biochemical recurrence, total cost of hospitalization, and cost of intraoperative consumables. The drainage tube was removed when the pelvic drainage fluid was below 20 ml per day. The catheter was removed when clear liquid was observed. Biochemical recurrence was defined as two consecutive postoperative PSA values greater than 0.2 ng/ml. Continence was defined as no urinary incontinence or needing fewer than 1 security pad per day. Docking time was defined as the time from the placement of the trocar to the moment at which all the robotic arms were successfully connected to the trocar. We used the whole-mount section technique to improve the positive margin detection rate.

All the data were analyzed using the SPSS 26.0 software package (version 26, IBM SPSS Statistics, Armonk, NY). The independent sample Student's t-test was used for the continuous variables in accordance with a normal distribution, which is reported as the mean and standard deviation (SD). Continuous variables that did not conform to a normal distribution were analyzed by the Mann–Whitney U test, which is reported as the median and interquartile range. Categorical variables were tested by Pearson’s chi-square test or Fisher’s exact test. Values of P < 0.05 were considered statistically significant.

## Results

All patients were successfully operated on, and no conversions to open surgery were needed. The demographic characteristics and preoperative clinical features of the two groups were similar and comparable (Table 1).

**Table 1:** Demographic characteristics and preoperative clinical features of the two groups of patients

Variables	Xi (n = 82)	Si (n = 93)	p value
Age, median (range), year	67.2 (50, 82)	68.1 (51, 85)	0.415
BMI, median (range), kg/m^2^	22.6 (18.6, 29)	22.1 (19, 29)	0.075
Diabetes mellitus, n (%)	8 (9.8)	11 (11.8)	0.809
Hypertension, n (%)	29 (35.4)	42 (42.5)	0.218
History of abdominal or pelvic surgery, n (%)	15 (18.3)	20 (21.5)	0.706
ASA score < 3, n (%)	72 (87.8)	81 (87.1)	1.000
ASA score ≥ 3, n (%)	10 (12.2)	12 (12.9)	1.000
Preoperative PSA, mean (SD), ng/ml	59.6 (85.6)	47.6 (82)	0.347
Prostate volume, mean (SD), ml	36.4 (18.8)	35.4 (18.5)	0.734
Biopsy Gleason score, n (%)			
≤ 6	9 (11)	12 (12.9)	0.817
= 7	28 (34.1)	26 (28)	0.415
≥ 8	45 (54.9)	54 (58.1)	0.760
Clinical T stage, n (%)			
cT2a	15 (18.3)	21 (22.6)	0.575
cT2b	52 (63.4)	45 (48.4)	0.490
cT2c	10 (12.2)	17 (17.2)	0.399
cT3a	5 (6.1)	11 (11.8)	0.293
D’Amico risk classification, n (%)			
Low	1 (1.2)	1 (1.1)	1.000
Intermediate	21 (25.6)	17 (18.3)	0.273
High	60 (73.2)	75 (80.6)	0.281

*SD* standard deviation, *BMI* body mass index, *ASA* American Society of Anesthesiologists, *PSA* prostate-specific antigen

The perioperative characteristics of the 175 patients are shown in Table 2. The anesthesia time, operation time, docking time and bladder neck-urethral anastomosis time in the Xi group were shorter than those in the Si group, and the differences between the two groups were statistically significant **(**respectively, 268.8 min vs. 219.3 min, P = 0.001; 228.2 min vs. 259.6 min, P < 0.001; 7.4 min vs. 12.7 min, P < 0.001; 22.9 min vs. 24.3 min, P = 0.028)**.** In addition, the postoperative bed rest time of the Xi group was shorter than that of the Si group (2.2 d vs. 2.6 d, P = 0.002), and catheter removal occurred earlier in the Xi group than in the Si group (8.6 d vs. 9.7 d, P = 0.036). There were no significant differences in estimated blood loss, intraoperative blood transfusion volume, postoperative recovery time of intestinal function, postoperative pelvic drainage duration time or postoperative hospital stay duration between the two groups.

**Table 2:** Perioperative characteristics of the Xi and Si groups

Variables	Xi (n = 82)	Si (n = 93)	p value
Anesthetic time, median (range), min	286.8 (210, 480)	319.3 (175, 510)	0.001
Operation time, median (range), min	228.2 (155, 450)	269.6 (135, 400)	< 0.001
Docking time, median (range), min	7.4 (4, 14)	12.7 (6, 22)	< 0.001
Bladder neck-urethral anastomosis time, mean (SD), min	22.9 (3.8)	24.3 (4.5)	0.028
Estimated blood loss, mean (SD), ml	240.9 (139)	279.3 (215.2)	0.169
Intraoperative blood transfusion, n (%)	4 (4.9)	7 (7.5)	0.545
Postoperative bed rest time, median (range), day	2.2 (2, 3)	2.6 (2, 8)	0.002
Postoperative recovery time of intestinal function, mean (SD), day	2.8 (2)	2.8 (1.3)	0.974
Postoperative pelvic drainage duration time, mean (SD), day	6(3.1)	6.5 (2.8)	0.293
Indwelling catheter time, mean (SD), day	8.6 (3.3)	9.7 (3.5)	0.036
Postoperative hospital stay, mean (SD), day	8.5 (3.1)	9.2 (3.7)	0.221

*SD* standard deviation

The incidences of postoperative complications classified as Clavien < 3 and those classified as Clavien ≥ 3 were similar between the two groups, with no significant differences (P = 0.715 and P = 0.545, respectively). There were no significant differences in the incidence of postoperative urethral stricture, PSM or biochemical recurrence between the two groups (P = 1.000, P = 0.445, and P = 1.000, respectively). Both groups showed good postoperative continence, and there were no significant differences in postoperative continence at the first, third and sixth months (P = 0.757, P = 1.000, and P = 0.772, respectively). The total cost of hospitalization in the Xi group was significantly higher compared the Si group (84,740.7 ¥ vs. 76,739.1 ¥, P = 0.003). In addition, there was a significant difference in the cost of intraoperative consumables between the two groups (13,199.4 ¥ vs. 10,823.0 ¥, P = 0.019). All the above data in this paragraph are displayed in Table 3.

**Table 3:** Complications, long-term outcomes and costs of the Xi and Si groups

Variables	Xi (n = 82)	Si (n = 93)	p value
Postoperative complications, n (%)			
Clavien < 3	17 (20.7)	22 (23.7)	0.717
Clavien ≥ 3	4 (4.9)	7 (7.5)	0.545
Urethral stricture, n (%)	8 (9.8)	9 (9.7)	1.000
Continence, n (%)			
First month	51 (62.2)	55 (59.1)	0.757
Third month	72 (87.8)	82 (88.2)	1.000
Sixth month	77 (93.9)	86 (92.5)	0.772
Follow-up, mean (SD), month	8.7 (1.7)	8.2 (1.7)	0.051
Positive margin, n (%)	44 (53.7)	56 (60.2)	0.445
Biochemical recurrence, n (%)	20 (24.4)	23 (24.7)	1.000
Total cost of hospitalization, mean (SD), ¥	84,740.7 (16,531.2)	76,739.1 (17,852.9)	0.003
Cost of intraoperative consumables, median (range), ¥	13,199.4 (4986, 34,121)	10,823 (5340, 20,784)	0.019

*SD* standard deviation

## Discussion

Radical prostatectomy generally includes transabdominal and extraperitoneal approaches, and each of these two techniques has its own advantages and disadvantages [7–9]. All 175 patients in this study underwent transperitoneal robot-assisted laparoscopic radical prostatectomy. In theory, the Xi system has been optimized, and the robotic arms have more joints, which should allow the surgeons to operate more smoothly, thus shortening the patients' time in the operating room. Schans et al. [10] compared 18 cases of partial nephrectomy with the Xi system and 18 cases of partial nephrectomy with the Si system and found that the docking time of the Xi system was significantly shorter than that of the Si system. Similar results have been demonstrated in a study of robot-assisted laparoscopic adrenalectomy [11]. In our study, the Xi system showed good docking ability, which is consistent with the results of the above studies, although this study focused on a different type of surgery from the above studies. In addition, a retrospective study on the application of the Xi system for colorectal cancer surgery showed that the operation time of the Xi system was significantly shorter than that of the Si system [12]. The results of several studies on the applications of the Xi system for urological surgery also showed that compared with the Si system, the former showed better results in terms of anesthesia time, operation time and docking time [13–15]. The above results are similar to the results of this study. We summarized the following reasons for these shortened times: first, because the docking of the Xi system is more convenient, the docking time is shortened; second, after the system was optimized, the operation fluency of the Xi system was also improved accordingly, so the operation time was shortened; and finally, the thinner robotic arms greatly reduce collisions between the robotic arms during the operation, which further speeds up the surgical process. In addition, the results of this study show that the indwelling catheter time in the Xi group was shorter than that in the Si group, which can be attributed to the excellent operation fluency and anastomosis ability of the Xi system.

Continence is one of the most important indicators for patients after radical prostatectomy and is directly related to patient quality of life after radical prostatectomy. The retention of continence mainly depends on the following factors: preservation of the neurovascular bundle, maximum preservation of the bladder neck and urethral length, and preservation of the puboprostatic ligaments [16]. Approximately 4% of patients who undergo radical prostatectomy need additional surgery to correct incontinence [17]. In this study, the continence rates in the first, third and sixth months after the operation in the Xi group were 62.2, 87.8 and 93.9%, respectively, which were not significantly different from those in the Si group. Urethral stricture is an important long-term complication after radical prostatectomy. Bladder neck-urethral anastomosis is one of the main factors affecting the occurrence of urethral strictures; therefore, the incidence of urethral strictures can indirectly reflect the quality of the anastomosis technique. In this study, although the time of bladder neck-urethral anastomosis in the Xi group was shorter than that in the Si group, there was no difference in the incidence of postoperative urethral stricture between the two groups, suggesting that the anastomotic effect was similar.

PSMs are an independent predictor of tumor progression. Some studies suggest that PSMs can be prevented by meticulous surgical techniques [18]. However, some scholars believe that PSMs are mainly related to the tumor stage and PSA value. The higher the stage and PSA value, the greater the probability of PSMs [19]. The PSM rate of robot-assisted laparoscopic radical prostatectomy reported in the literature ranges from 10.5 to 45.9% [3, 20]. In this study, the PSM rates in the Xi group and Si group were as high as 53.7 and 60.2%, respectively, but there was no significant difference between the two groups. We attribute the high PSM rate to the following reasons. First, high-risk patients accounted for 73.2 and 80.6% of patients in the Xi group and Si group, respectively, which increased the PSM rate in both groups. In addition, our hospital uses the whole-mount section technique, which further increases the PSM rate. Biochemical recurrence is another important problem for patients who undergo radical prostatectomy. Generally, biochemical recurrence after radical prostatectomy occurs 6–18 months earlier than clinical recurrence, so biochemical recurrence is believed to indicate clinical recurrence. After clinical recurrence, the patient survival time is significantly shortened [21]. There are many factors that affect the biochemical recurrence rate, but only PSMs can be avoided by surgeons. In this study, there was no significant difference in the biochemical recurrence rate between the two groups.

Although da Vinci robotic surgery systems (whether Xi or Si) surpass traditional laparoscopy in many ways and greatly improve the efficiency of surgeons, the utilization rate of laparoscopy still far exceeds that of robotic surgery systems worldwide, especially in small medical centers, mainly due to the high cost of purchasing and maintaining the da Vinci surgical system. In addition, most commercial and government insurance programs do not cover the cost of robotic surgery. Therefore, the cost to the patient will change accordingly. In our study, the total cost of hospitalization and the cost of intraoperative consumables in the Xi group were higher than those in the Si group. However, the cost of robotic surgery with the Xi system varies among medical centers and may be higher or lower than that with the Si system. Therefore, the cost of robotic surgery with the Xi system should not be generally considered higher than with the Si system [11, 14]. Nevertheless, the purchase and maintenance costs of the Xi system are higher than those of the Si system.

There were some limitations in our study. First, being a retrospective study, selection bias may be present. In addition, because the Xi system was introduced in our center for only a short time, the sample size of our study was low, which reduces the reliability of the results to some extent.

## Conclusions

In summary, our study shows that for radical prostatectomy, the perioperative outcomes with the Xi system are better than those with the Si system. Although both systems have similar oncology outcomes, the cost in the Xi group was higher compared to the Si group. In addition, long-term follow-up data are needed to prove whether a difference exists in postoperative long-term results between the two groups.

## Data Availability

The datasets used and/or analyzed in the present study are available from the corresponding author upon reasonable request.

## Abbreviations

BMI: Body mass index
ASA: American Society of Anesthesia
PSA: Preoperative prostate-specific antigen
PSM: Positive surgical margin
SD: Standard deviation
